# NK/T cell lymphoma-induced ectopic ACTH syndrome: a case report

**DOI:** 10.3389/fonc.2026.1745880

**Published:** 2026-03-23

**Authors:** Shengya Xu, Difei Lu, Xue Zhao, Jingcui Guo, Ying Gao, Junqing Zhang

**Affiliations:** Department of Endocrinology, Peking University First Hospital, Beijing, China

**Keywords:** adrenal gland mass, Cushing’s syndrome, ectopic ACTH syndrome, hematologic malignancies, Nk/t cell lymphoma

## Abstract

**Background:**

Primary adrenal natural killer (NK)/T cell lymphoma is extremely rare with rapidly aggressive clinical manifestation and poor prognosis. Here, we report a case of NK/T-cell lymphoma with bilateral adrenal involvement and secondary ectopic adrenocorticotropin syndrome (EAS).

**Case presentation:**

This is a 56-year-old woman with main complaint of fatigue and slight weight loss. Bilateral adrenal mass, rapid progress of pancytopenia, and elevated cortisol and ACTH levels were discovered. Cushing’s syndrome was diagnosed when serum cortisol was not suppressed after 1-mg dexamethasone suppression test and low-dose dexamethasone suppression test (LDDST). Morning cortisol and ACTH levels markedly increased after 1 week, whereas the pathology of bone marrow revealed NK/T cell lymphoma, which indicated the diagnosis of EAS. After two cycles of chemotherapy, the patient died 6 months after diagnosis.

**Conclusion:**

NK/T cell lymphoma should be considered in the differential diagnosis of bilateral enlarged adrenal mass and could induce ectopic ACTH syndrome. For rapid progressing malignancy, the clinical features of Cushing’s syndrome may be absent in patients with EAS.

## Background

Ectopic adrenocorticotropin syndrome (EAS), which is induced by a wide spectrum of aggressive malignancies, accounts for 10% of patients with Cushing’s syndrome (1). As a specific kind of paraneoplastic syndrome, EAS is commonly secondary to malignant tumors including small cell lung carcinoma (SCLC), bronchial neuroendocrine neoplasms (NEN), and pheochromocytoma. Primary natural killer (NK)/T cell lymphoma of adrenal glands is an extremely rare malignant disease with poor clinical prognosis, and only a few cases were reported worldwide. To date, no case of EAS induced by lymphoma was reported. Here, we present a case report of EAS secondary to NK/T-cell lymphoma with bilateral adrenal involvement.

## Case presentation

**Figure 1:**
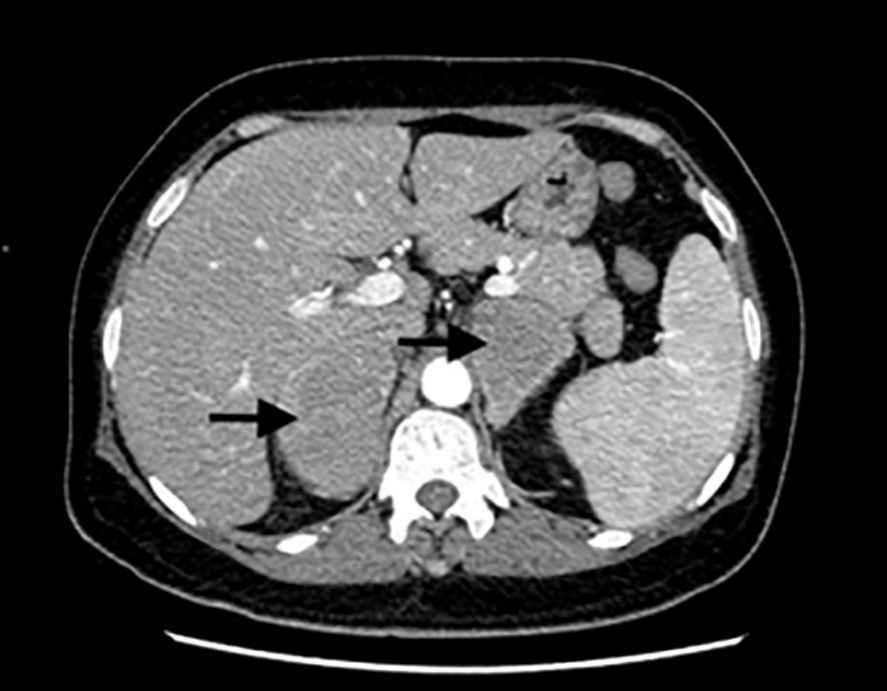
Abdominal CT scans indicated bilateral adrenal mass (noted by two black arrows). Enhanced CT showed bilateral adrenal mass with mild cord-like enhancement and slightly enlarged spleen.

A 56-year-old female experienced fatigue and slight weight loss 1 month before she turned to outpatient clinic for help. She underwent abdominal contrast-enhanced computed tomography (CT) scan, and bilateral adrenal mass with the maximal size of 3.9 × 3.5 cm and mild cord-like enhancement in the right adrenal gland was discovered (**Figure 1**). Her blood pressure and glucose level remained normal. Body height was 150 cm, body weight was 55 kg, and body mass index (BMI) was 24.4 kg/m^2^. Blood pressure was 120/68 mmHg, and clinical symptoms including purple striae, supraclavicular fatty pad, buffalo hump, purpura, or superficial lymphadenopathy were not noticed in the patient.

**Figure 2:**
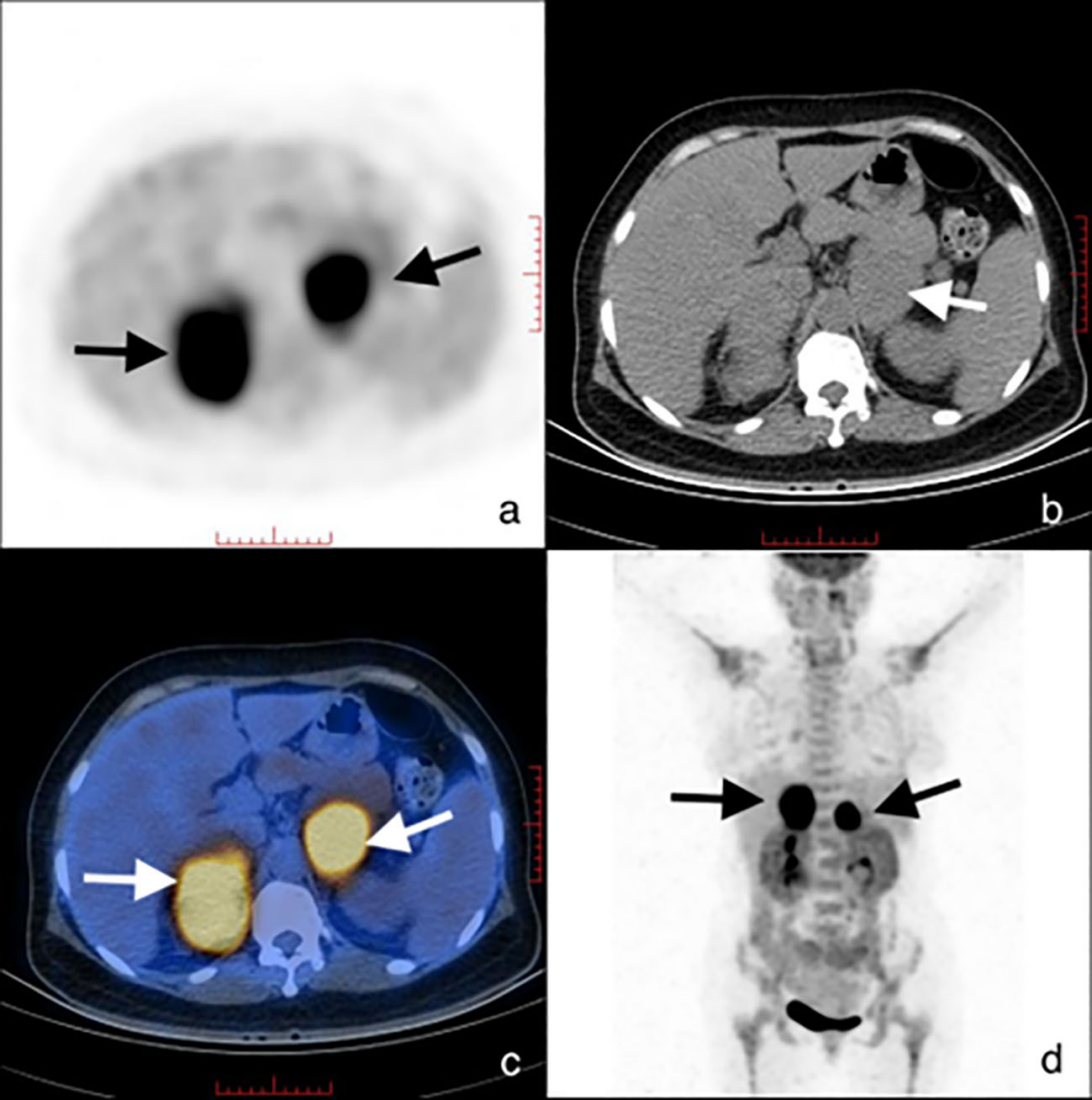
^18^F-FDG PET image **(a)**, transaxial CT **(b)**, PET-CT image **(c)**, and torso PET image **(d)** revealed uptake in bilateral adrenal glands with SUV_max_ of 17.9 (noted by arrows). SUV, standard uptake value.

After she was admitted, laboratory tests showed pancytopenia: white blood cell count (WBC) 3.5 × 10^9^/L, hemoglobin 89 g/L, platelet 61 × 10^9^/L, neutrophil percentage 67.8%, and eosinophil count 0.0 × 10^9^/L. The pancytopenia aggravated after 1 week: WBC 2.6 × 10^9^/L, hemoglobin 69 g/L, platelet 14 × 10^9^/L. Her glucose level was in the normal range, while mild hypopotassemia was noticed (3.35 mmol/L). Her HCO_3_^−^ level was 22.6 mmol/L (normal range: 22-30), her lactate dehydrogenase (LDH) level was 431 U/L (normal range: 100-240), and her hydroxybutyrate dehydrogenase (HBDH) level was 320 U/L (normal range: 90-220). Her cortisol rhythm was abnormal, and the cortisol level was not suppressed after 1-mg dexamethasone test and low-dose dexamethasone suppression test (LDDST) (**Table 1**). Her 24-h urine cortisol level was 4470.0 μg/24 h (normal range: 370-639), and it was not suppressed after LDDST (2,337.3 μg/24 h). Her early morning cortisol and ACTH levels were elevated even within the first week after LDDST (morning cortisol 49.94 μg/dL, ACTH 118 pg/mL). The above tests indicated ACTH-dependent Cushing’s syndrome. The plasma renin, aldosterone, and metanephrine levels were all in normal ranges, thus primary aldosteronism and pheochromocytoma were ruled out. Positron emission tomography (PET)/CT revealed increased glucose uptake in bilateral adrenal glands, bilateral humerus, axial bone, sacrum, and iliac bone and thickened intestinal wall in the descending segment of duodenum (**Figure 2**).

**Table 1:** Cortisol and ACTH levels of the patient.

Hormone	8 am	4 pm	0 am	1 mg Dex	LDDST*
Cortisol (μg/dL) (Normal range: 6.2-19.4)	25.74	25.88	23.07	31.45	38.76
ACTH (pg/mL) (Normal range: 7.2-63.3)	10.40	14.95	15.99	13.25	117.4

ACTH, adrenocorticotropin; Dex, dexamethasone; LDDST, low dose dexamethasone suppression test. ACTH and cortisol were measured using chemiluminescent immunoassay.

* LDDST was performed 2 days after 1-mg dexamethasone suppression test.

**Figure 3:**
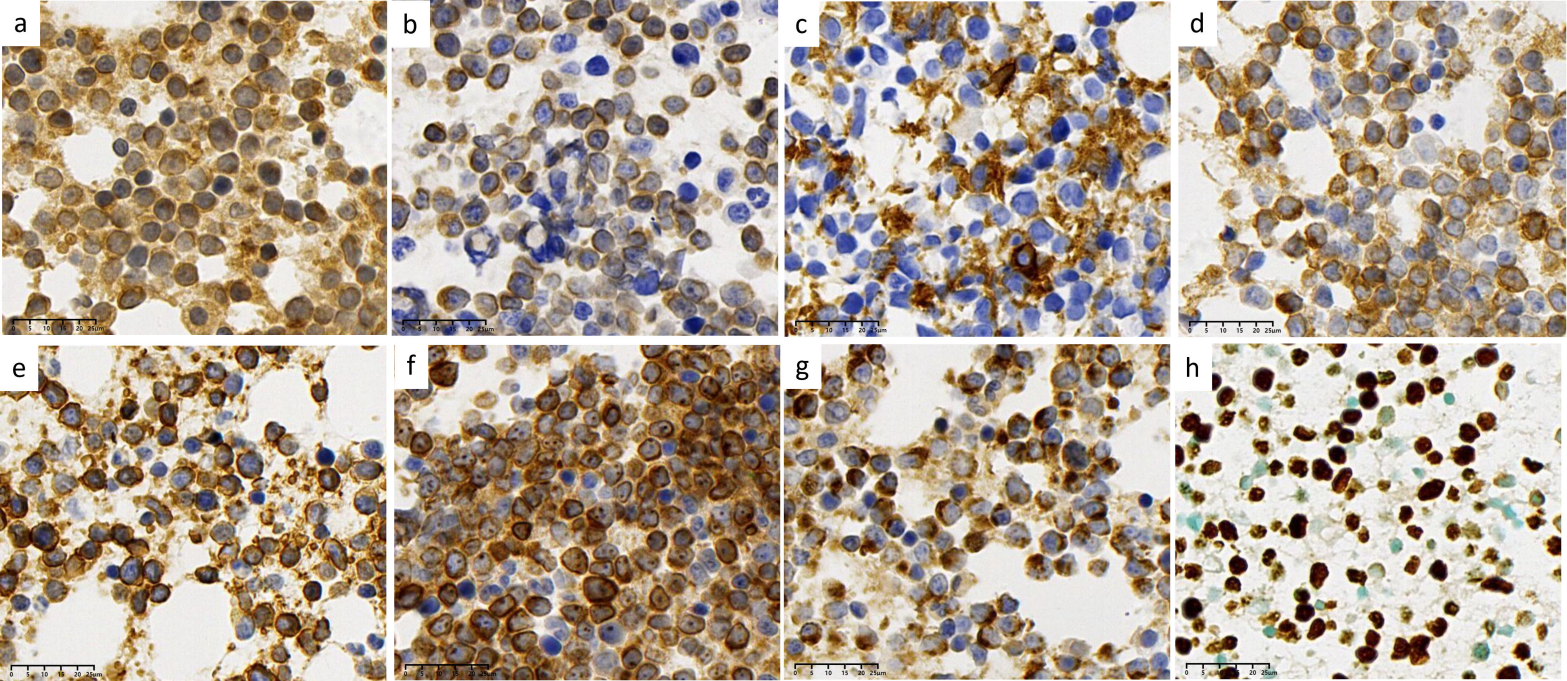
Histological and immunohistochemical analysis of bone marrow specimens, positive (magnification × 80) for CD2 **(a)**, CD3 **(b)**, CD4 **(c)**, CD7 **(d)**, CD56 **(e)**, Bcl-2 **(f)**, TIA-1 **(g)**, and EBER **(h)**.

The cytology of bone marrow aspiration was performed due to pancytopenia and showed 57% lymphocyte-like protoblasts with negative POX, NSE, and PAS, indicating the suspected diagnosis of lymphoma cell leukemia. The pathology specimens of bone marrow suggested diffused infiltration of small to medium heterotypic neoplastic lymphoid cells. Immunohistochemistry analysis showed that the tumor cells were positive for CD3, CD56, CD7, CD2, CD4, Bcl2, TIA-1, and EBV-encoded RNA (EBER) (**Figure 3**), negative for TdT, CD20, and CD8. The Ki67 labeling index was 90%. The serum level of EBV-DNA was 3.43 × 10^6^ copies/mL.

She was diagnosed with NK/T-cell lymphoma with bilateral adrenal involvement and EAS. Hemophagocytic syndrome was diagnosed based on the symptoms of fever (Tmax 38.7 °C), elevated ferritin (1,395 ng/mL), soluble CD25 (32,375 pg/mL), and reduced NK cell activation (17.5%). She received two cycles of chemotherapy with DEP+L (etoposide 150 mg d1, d8, d15, d 22; doxorubicin 40 mg d7; dexamethasone 2 mg d22-d28; asparagine 3,750 IU d13). At the 6-month follow-up, the bone marrow aspiration of the patient remained 3.0% of protoblasts, and a 2-cm lesion in the central nervous system was discovered in brain magnetic resonance imaging (MRI), which was absent at the first diagnosis. Thereafter, the patient refused to continue the chemotherapy and died 4 weeks later.

## Discussion

Here, we report a female patient whose main manifestation was bilateral adrenal mass and pancytopenia. After evaluation of adrenal endocrine function, the diagnosis of ACTH-dependent Cushing’s syndrome was established. Accompanied by rapidly progressed malignancy, the ACTH and cortisol levels significantly increased, indicating the diagnosis of EAS. To the best of our knowledge, our case is the first to report NK/T-cell lymphoma-induced EAS with bilateral adrenal involvement.

In terms of the diagnosis of EAS, both 1-mg dexamethasone suppression test and LDDST (48 h, 2 mg/day) were performed in our patient to confirm endogenous hypercortisolism. Due to the rapid progression of the lymphoma and negative result in the pituitary using brain MRI and PET/CT, neither laboratory analysis for the differential diagnosis of Cushing’s syndrome including bilateral inferior petrosal sinus sampling (BIPSS) nor high-dose dexamethasone suppression test (HDDST) was arranged for the patient (2). The 24-h urinary free cortisol exceeded 10× the upper limit of normal (ULN) in our case, which provided strong evidence for the diagnosis of EAS with sensitivity of 80% and specificity of 94% (3). Patients with EAS commonly demonstrate unchanged serum and urinary cortisol levels to the HDDST, while approximately 20% of EAS presents with either serum or urinary cortisol suppression after the HDDST, which brings about difficulty in the differential diagnosis with Cushing’s disease (CD) (4). Limited clues were obtained for the confirmation of EAS in our case. The rapidly deteriorating clinical picture in the absence of Cushingoid features, accompanying hypokalemia, normal brain MRI finding at diagnosis, significantly increased ACTH and cortisol levels, and lymphoma worsening supported the diagnosis of EAS.

In comparison to CD, EAS is a rare condition of ACTH-dependent Cushing’s syndrome. EAS induced by carcinoid is slowly progressed, and common Cushingoid appearance could present. However, EAS induced by tumors with high malignancy may not present typical Cushingoid appearance; instead, hypokalemia, alkalemia, and other consumptive symptoms as well as rapid deterioration of clinical manifestations are the common manifestations (5). Our patient presented with hypokalemia and consumptive symptoms, and no Cushing’s features were noticed due to rapid development of the malignancy.

The manifestations of EAS are slightly distinguished from CD. In a retrospective study of 88 cases of EAS, 45.9% cases appeared with purple striae, 76.5% with supraclavicular fatty pad, and 57.8% with weight gain, and the risk of above symptoms was significantly lower compared with CD patients (6). On the contrary, weight decrease was reported in 21.8% patients with EAS, while none of the CD patients experienced weight loss (6). In terms of our case, the absence of Cushingoid appearance, hypertension, or increased glucose level may be explained by the rapid progression of NK/T cell lymphoma. Weight loss and fatigue resulted by NK/T cell lymphoma and pancytopenia were predominant before the clinical features of Cushing’s syndrome could appear. Additionally, in accordance with our case, the proportion of hypopotassemia in EAS patients (72.1%) was significantly higher compared with CD patients (19.1%) (6). In EAS patients with hypokalemia, the mean level of serum cortisol was 42.05 μg/dl, the ACTH level was 186.0 pg/ml, and the 24-h urinary cortisol level was 1,169.69 μg/24 h (4), which increased the similar extent compared with our case.

The diagnosis of the causes of ectopic ACTH production remains challenging. The main causes of EAS include pulmonary or pancreatic neuroendocrine tumors, pulmonary carcinoid, medullary thyroid carcinoma, and pheochromocytoma (7). It is a common clinical situation that CT or MRI fails to provide enough evidence on the cause of EAS. In the past decade, the implication of molecular imaging including ^18^F-fluorodeoxyglucose (FDG) or ^68^Ga-DOTA-Tyr3-octreotate (DOTATATE)-PET/CT ameliorated the diagnose rate of the primary tumor of EAS to a large extent, particularly for the diagnosis of carcinoid by ^68^Ga-DOTATATE PET/CT (8). In our case, pancytopenia and PET/CT provided critical evidence for bone marrow biopsy and confirmed diagnosis of NK/T cell lymphoma.

Our patient was presumptively diagnosed with NK/T cell lymphoma with predominant adrenal mass when a lack of lymphadenopathy was noticed at the first presentation. Extra-nodal NK/T cell lymphoma is rarely presented in adrenal glands. Primary adrenal lymphoma is mainly diffuse large B-cell lymphoma in histopathology (9); thus, adrenal glands being the initial presentation is extremely rare. To date, eight cases of primary adrenal NK/T cell lymphoma including our case were reported (10–16). The age of the cases ranged from 17 to 79 years, and the male to female ratio was 5:3. 50% of the patients including our case presented with B symptoms (fever, weight loss, and sweating). 75% of the cases (6/8) presented with bilateral adrenal mass, and the max tumor diameter ranged from 3.1 to 13.8 cm. Bone marrow involvement was reported in three patients (3/8, 37.5%) including our case. The prognosis of these patients was poor, and the survival duration was 3 months to 2 years after diagnosis under chemotherapies.

To the best of our knowledge, no previous case of NK/T cell lymphoma induced EAS was reported. The mechanism of EAS and the source of ectopic ACTH production remained unclear. Researchers discovered that the ACTH level was paradoxically elevated after dexamethasone suppression test in a case of pheochromocytoma causing EAS, which was in accordance with our case, while metyrapone administration could decrease both ACTH and cortisol levels (17), which suggested a glucocorticoid-driven positive-feedback regulation in EAS. Takeuchi et al. performed a negative immunostaining for ACTH in the tumor tissue of prostate small cell carcinoma causing EAS, while the proopiomelanocortin (POMC) mRNA expression in the primary lesion was positive (18). A possible hypothesis for the phenomenon was that aberrant POMC also produced an anti-ACTH antibody and could prevent immunodetection for ACTH, and the synthesis of ACTH produced by tumor cells was low but rapidly secreted. In a recent study, a novel cell type with high expression of POMC and c releasing hormone (CRH) called ACTH+ and CRH+ pheochromocyte was discovered in a case with ectopic ACTH- and CRH-secreting pheochromocytoma, indicating that ectopic CRH release contributed to the development of EAS (19). The limitation of our study is the lack of immunostaining in adrenal mass of the patient due to high hemorrhage risk caused by pancytopenia; otherwise, the mechanism of EAS in NK/T cell lymphoma could be partially explored. In addition, the lack of HDDST or BIPSS due to increased risk of hemorrhage and infection weakened the diagnostic certainty of EAS.

## Conclusion

In conclusion, NK/T cell lymphoma should be considered in the differential diagnosis of bilateral enlarged adrenal mass and could induce ectopic ACTH syndrome. For rapid progressing malignancy, the clinical features of Cushing’s syndrome may be absent in patients with EAS.

## Data Availability

The original contributions presented in the study are included in the article/supplementary material. Further inquiries can be directed to the corresponding author.
